# Measuring self-as-context in Chinese college students: Validity and reliability of the Chinese version of self-as-context scale (C-SACS)

**DOI:** 10.3389/fpsyg.2022.1051661

**Published:** 2022-12-06

**Authors:** Shuanghu Fang, Mingjie Huang, Yiyi Wang

**Affiliations:** ^1^School of Educational Science, Anhui Normal University, Wuhu, China; ^2^Department of Psychology, University of Toronto Mississauga, Missisauga, ON, Canada

**Keywords:** acceptance and commitment therapy (ACT), psychological flexibility, self-as-context, validity, reliability, college students

## Abstract

**Objective:**

To examine the validity and reliability of the Chinese version of the Self-as-Context Scale (SACS) in college students.

**Method:**

We used convenience sampling to recruit 708 Chinese college students. All participants completed the SACS and 343 of them were asked to complete the validation questionnaires (Satisfaction with Life Scale, Peace of Mind Scale, Acceptance and Action Questionnaire-II, Mindful Attention Awareness Scale, Cognitive Fusion Questionnaire-Fusion, and Depression Anxiety Stress Scale-21) at the same time. We conducted items analysis, exploratory factor analysis (EFA), confirmatory factor analysis (CFA), measurement invariance test, correlation analysis, regression analysis, and internal consistency reliability analysis. After 3 weeks, 217 participants filled out the SACS again to assess the test–retest reliability.

**Results:**

The exploratory factor analysis showed that the SACS consisted of two factors (Centering and Transcending), with a total of 9 items. The confirmatory factor analysis demonstrated that the two-factor structure fit well (*χ*^2^ = 55.40, *df* = 22, CFI = 0.977, TLI = 0.963, RMSEA = 0.065, SRMR = 0.032). According to the results of the measurement invariance tests, configural invariance, metric invariance, scalar invariance, and strict invariance of the 2-factor model, the C-SACS scores were comparable across genders. Additionally, the C-SACS total score and its subscale scores were significantly positively correlated with positive indicators of mental health (life satisfaction, affective well-being), significantly negatively correlated with negative emotions (depression, anxiety, stress), significantly negatively correlated with experiential avoidance and cognitive fusion (except for the Transcending factor), and significantly positively correlated with mindful attention and awareness. Regression analysis results revealed that the C-SACS surpasses the incremental effectiveness of AAQ-II and CFQ-F in predicting different psychological health indicators. The Cronbach’s *α* coefficients of the C-SACS and two subscales were 0.88 [0.71, 0.90], 0.80 [0.87, 0.90] and 0.85 [0.83, 0.88] and McDonald’s *ω* = 0.88 [0.87, 0.90], *ω* = 0.80 [0.78, 0.83], *ω* = 0.85[0.83, 0.88]. The test–retest reliability (ICC) was 0.73 and 0.72, respectively.

**Conclusion:**

The results of our study suggest that the Chinese version of SACS has good reliability and validity in Chinese college students.

## Introduction

Acceptance and commitment therapy (ACT), a third-wave cognitive-behavioral therapy (CBT), has gained increasing attention from researchers due to its efficacy in chronic pain, depression, anxiety, stress, addiction, and other mental health problems with different populations (Hayes, 2004; A-Tjak et al., 2015; Fang and Ding, 2020a,b). Psychological flexibility, a core component of ACT, has also gained much attention. ACT aims to improve individuals’ psychological flexibility, which can help individuals to fully immerse in the present moment, face and accept their internal experiences (e.g., thoughts, emotions, body sensations, memories, motivation, personal experiences, etc.) without any defense, allow thoughts and emotions to be their original formats, make decisions, insist or change behaviors based on their internal values, and commit to a valuable and meaningful life (Hayes, 2016; Fang and Ding, 2020a). Psychological flexibility consists of six components: cognitive defusion, acceptance, self as context, contact with the present moment, values, and commitment to action (Hayes, 2016; Fang and Ding, 2020a). Nowadays, as research moves toward process-based therapies and a new generation of evidence-based care targeting core mediators and moderators (Hofmann and Hayes, 2019), there is a growing demand to study the role of six processes of psychological flexibility and their roles in the mechanisms of change (Hofmann and Hayes, 2019; Ren et al., 2019). Thus, tools that can accurately measure six components of psychological flexibility have become critically valuable.

Currently, the six processes of psychological flexibility have corresponding measurement tools in China except for self-as-context (Cao et al., 2013; Zhang et al., 2014; Wong et al., 2016; Bi et al., 2021; Cao et al., 2021; Li et al., 2021). Self-as-context refers to the ability to observe one’s thoughts and feelings from an observant perspective and to deal with negative thoughts calmly and flexibly (Hayes, 1984; Hayes et al., 2012; Zhang et al., 2012). Self-as-context, also known as the transcendent or observing self (Moran et al., 2018), is the ability to observe private events such as emotions, thoughts, and memories and learn that “I am more than my experiences” (Hayes, 2016). It allows individuals to focus on a stable state of self that is separate from, or more than, internal thoughts and experiences (Barnes-Holmes et al., 2001). Self-as-context is different from other ACT components, or it can be considered as the carrier of the other five components: (1) from the perspective of the relational framework theory, self-as-context is one way of perspective taking, a special kind of municipal relationship, which is here and now; (2) it focuses on helping people understand that experiences, thoughts, and feelings are only transcendent—they do not fundamentally impact the core self. Therefore, self-as-context not only has an important impact on individual self-concept and personality development (Peng et al., 2012; Peng, 2018) but also is an essential part of evaluating the effect of ACT intervention and exploring the change mechanism. Some studies revealed that self-as-context is closely related to people’s well-being and psychological health (depression, anxiety, and, stress; Chen, 2013; Zettle et al., 2018; Morgan and Aljabari, 2019).

The concept of self-as-context is particularly in line with Chinese culture, as it is often expressed in ancient Chinese sayings such as “I examine myself three times every day” (Ames and Rosemont Jr, 2010) and “with the sky as a cover…the earth as a public transport…and the four seasons as horses…” (Chen, 2021). The observing self emphasizes observing one’s own emotions from an observational perspective and different angles while establishing a connection with oneself. The Chinese traditional culture encourages individuals to jump out of the situation and focus on their real experiences from an observational perspective, promoting the dissociation from negative thoughts and emotions to accept them (Xie, 2021). With the increasing rise of ACT research in China and the research’s devotion to the efficiency of process-based therapies, measurement tools have become essential for examining the level of self-as-context. The relevant tool not only provides empirical data for assessing the level of self-as-context development in the Chinese population but also can provide important indicators for assessing the effectiveness of process-based intervention packages. The previous studies in China showed that self-as-context plays an important role in the mental health, well-being, self-concept, and personality development of college students (Peng et al., 2012; Chen, 2013; Peng, 2018). These studies also showed that the intervention of self-centered technology significantly improved college students’ depression, anxiety, and stress.

As a major individual psychological function, self-as-context should be measured in different populations or fields. Nevertheless, the commonly used measurement tool, the Self Experiences Questionnaire (SEQ; Yu et al., 2016), was only based on patients with chronic pain. Since the development and evaluation of SEQ were limited to patients with chronic pain, the applicability of SEQ could not be generalized to other populations. Zettle et al. (2018) developed the Self-as-context Scale (SACS) using clinical samples and non-clinical samples and discussed the effectiveness and reliability of the tool. The SACS is a two-dimensional structure, including Centering and Transcending. Centering reflects the individual’s ability to calmly deal with unwanted thoughts and feelings, while Transcending reflects the type of invariant perspective-taking of the observational self, to perceive events objectively without being disrupted by internal negative thoughts and feelings. In addition to good internal consistency and excellent convergence validity, it reveals the incremental effectiveness of SACS in predicting well-being (satisfaction with life) and general mental health (GHQ). Compared to SEQ, SACS includes fewer items and is more cost-effective to use. Rather than limiting to specific groups and fields, SACS generally measures individuals’ ability to truly perceive and respond flexibly to their thoughts or feelings. Zettle et al. (2018) revealed that SACS scores can better distinguish between clinical and non-clinical samples. Therefore, it has received massive attention from researchers and clinicians and has been used to measure self-as-context in ACT studies and interventions for different populations, such as students (Moran et al., 2018), law enforcers (Baker et al., 2020), and people with depression and anxiety (Østergaard et al., 2020). The above studies suggest that SACS, as an effective measurement tool for self-as-context, has a wide range of scientific research application value. However, in the original study, all participants were a convenience sample of college students, except for a small clinical sample. The authors concluded that the extent to which the two-dimensional structure of the scale extends to other populations is an empirical question (Zettle et al., 2018). This can only be adequately addressed by further research with other samples (Zettle et al., 2018).

The present study recruited Chinese college students as participants, aiming to explore the validity and reliability of the Chinese version of SACS among Chinese populations. We examined the C-SACS’s structural validity, criterion validity, and internal consistency reliability. Criterion validity was examined by using the Acceptance and Action Questionnaire–II (AAQ-II; Cao et al., 2013), Mindful Attention Awareness Scale (MAAS; Chen et al., 2012), Cognitive Fusion Questionnaire-Fusion (CFQ-F; Zhang et al., 2014), Depression Anxiety Stress Scale-21 (DASS-21, Gong et al., 2010), Satisfaction with Life Scale (SWLS; Xiong and Xu, 2009) and Peace of Mind Scale (POM; Lee et al., 2013). Previous studies revealed that self-as-context is closely correlated with the other five processes and well-being (Hayes et al., 2012; Zhang et al., 2012). For example, self-as-context is inextricably linked with other processes, and the core goal of the six core technologies is to improve people’s psychological flexibility, and the ultimate goal is to promote people to live a valuable and meaningful life. So, the AAQ-II, MAAS, CFQ-F, SWLS, and POM were included to examine the criterion validity of the SACS. In addition, since previous studies revealed that self-as-context has a considerable impact on students’ negative emotions (depression, anxiety, and stress, Chen, 2013), the DASS-21 was also tested. Our specific goals were as follows: (1) to explore whether the Chinese version of SACS indicates the same factor structure as in the original version; (2) to determine whether SACS is correlated with Chinese college students’ general psychological health and psychological distress; (3) to examine the reliability and validity of the C-SACS among Chinese college students.

## Materials and methods

### Participants

This study was approved by the Ethical Committee of Anhui Normal University. All students voluntarily participated in the research and none had significant clinical psychological symptoms. All participants were treated under the Declaration of Helsinki and its latest amendments and were provided with written informed consent before participating in the study. Parents of participants who were under 18 years old were informed and their consent was obtained. The eligibility criteria for participants were as follows: (1) They had registered university status; (2) They had studied in a university for more than 6 months.

In this study, a convenience sampling method was used and the data were obtained through an Internet applet (sojump-Wenjuanxing). The first author of this paper contacted the student administration of three universities (from Beijing, Hefei, and Wuhu), explained to them in detail the purpose of the study and the research procedures, and obtained their consent. The first author of this study conducted online training for the counselors involved in the study and then forwarded the questionnaire link to the counselors, who explained the study procedures in detail to the students. Written consent was obtained from each participant before completing the questionnaire (online document collection applet). After submitting the questionnaire, participants received a certain reimbursement.

Two rounds of data collection were conducted: the first batch of respondents filled out both SACS and the validation questionnaires (*N* = 369) and 343 of them were valid; the second batch of respondents only filled out the SACS questionnaire (*N* = 397) and 365 of them were valid. In total, we received 766 SACS responses. To prevent participants from answering randomly without paying attention, 3 catch questions were included in the survey. After excluding invalid questionnaires (i.e., missing one out of three), a total of 708 valid SACS responses were obtained with a retention rate of 92.40%. There were 158 males and 550 females, aged between 17 and 25 years old (Mean = 20.28). 377 of the participants were freshmen, 96 were in their second year, 210 were in their third year, and 25 were in their fourth year. 344 of them major in science and engineering, 186 of them major in literature and history, and the rest major in other domains. 429 students were from rural areas and 279 from urban areas. Three weeks later, the respondents who only filled out the SACS questionnaire were invited to fill out the SACS again to examine the test–retest reliability. 226 questionnaires were obtained and 217 of them were valid with a retention rate of 96.01%.

### Procedure

The first author of this study contacted the original author of the questionnaire, and with the consent of the original author, we obtained the right to translate and revise the SACS. The process of translation involves translating from English to Chinese (forward translation; World Health Organization, 2020) and Chinese back to English (back translation; Erkut, 2010). First, the first author of this paper, a native speaker of Chinese and fluent in English, translated the SACS into Chinese and modified ambiguous terms to get the first draft of the Chinese version. Secondly, two other psychologists working in English-speaking countries translated the Chinese version draft back into English. By taking into account the opinions of two experts, we obtained the back-translated English version of the scale. Next, the five experts who participated in the translation and back-translation formed an expert group to discuss and compare the differences between the original English version, the English version after back-translation, and the first draft of the Chinese version through multiple online meetings. They revised and modified the first draft of the Chinese version and obtained the second draft of the Chinese version of the scale. Furthermore, we randomly invited twenty undergraduates and postgraduates in non-psychology majors from a university library or a self-study room to evaluate and provide feedback on the readability and comprehensibility of each item of the Chinese version of the second draft. After comprehensively considering relevant evaluations and suggestions, the first author of this paper modified and reviewed the Chinese version of SACS again, and obtained the final Chinese version of SACS (see Appendix 1). The scoring of this Chinese version was consistent with the original scale.

### Measures

The Self-as-Context Scale (SACS) was developed by Zettle et al. (2018) to measure the contextual self in two domains, Centering and Transcending. Centering reflects the individual’s ability to calmly deal with unwanted thoughts and feelings, while Transcending reflects the type of invariant perspective-taking of the observational self, to perceive events objectively without being disrupted by internal negative thoughts and feelings. There are 10 items on the scale, and each item is scored on a 7-point Likert scale (1 = strongly disagree to 7 = strongly agree). Higher scores indicate a higher self-as-context level.

#### The satisfaction with life scale – Chinese version

The Satisfaction with Life Scale (SWLS) is a 5-item scale developed by Diener et al. (1999) to measure individuals’ life satisfaction. The original version was translated into Chinese by Xiong and Xu (2009). Participants were asked to rate the extent to which they agree or disagree with each item on a scale ranging from 1 (strongly disagree) to 7 (strongly agree). Higher scores indicate higher life satisfaction (subjective happiness). The scale has been widely used to measure individual well-being since its revision. The Cronbach’s alpha was 0.86 in our sample, suggesting good internal consistency.

#### The peace of mind scale – Chinese version

The Peace of Mind Scale (POM) was compiled by Taiwan scholar Lee et al. (2013) to measure an individual’s calm and harmonious state of mind. It has a total of 7 items, with each item being rated on a 5-point Likert scale, ranging from 1 (never) to 5 (always). Higher scores represent higher levels of inner peace and harmony. The Cronbach’s alpha was 0.88 in our sample, indicating good internal consistency.

#### The acceptance and action questionnaire–II – Chinese version

The Acceptance and Action Questionnaire–II (AAQ-II) is a 7-item scale developed by Bond et al. (2011) to assess individuals’ experiential avoidance. It was translated into Chinese by Cao et al. (2013). Participants were asked to rate on a scale of 1 (never true always) to 7 (always true) how frequently they have each experience. Higher scores reflect higher experiential avoidance. The Cronbach’s alpha was 0.92 in our sample, suggesting excellent internal consistency.

#### The mindful attention awareness scale – Chinese version

The Mindful Attention Awareness Scale (MAAS) is a 15-item scale developed by Brown and Ryan (2003) to assess individuals’ receptive awareness and attention to what is happening in the present moment. It was translated into Chinese by Chen et al. (2012). Participants were asked to rate on a scale of 1 (almost always) to 6 (almost never) how frequently they have each experience. Higher scores reflect higher levels of mindful awareness at that moment. The Cronbach’s alpha was 0.88 in our sample, suggesting good internal consistency.

#### The cognitive fusion questionnaire-fusion – Chinese version

The Cognitive Fusion Questionnaire-Fusion (CFQ-F) is a 9-item scale developed by Gillanders et al. (2014) to measure individuals’ degrees of cognitive fusion. It was later translated into Chinese by Zhang et al. (2014). Each item is rated on a 7-point Likert scale (1 = obviously inconsistent to 7 = obviously consistent). Higher scores indicate higher levels of cognitive fusion. In our sample, Cronbach’s was 0.94, suggesting excellent internal consistency.

#### Depression anxiety stress scale-21 – Chinese version

The Depression Anxiety Stress Scale-21 (DASS-21) is a 21-item scale developed by Lovibond and Lovibond (1995) to assess individuals’ individuals’ depression, anxiety, and stress levels (Antony et al., 1998). It was translated into Chinese by Gong et al. (2010). Responses were collected on a scale of 0 (did not apply to me at all) to 3 (applied to me very much or most of the time) with higher scores indicating higher emotional difficulties. The Cronbach’s alpha of the total scale and its depression, anxiety, and stress subscales in the study were 0.94, 0.87, 0.84, and 0.85, respectively.

### Statistical analysis

Statistical analysis was conducted using SPSS22.0, Mplus7.4 and R 4.0.5. First, with the use of the SPSS random number generator and a fixed value of 2,021,626 as the starting point, we randomly generated numbers in the range of 1–1,000, and randomly divided the entire sample (*N* = 708) into 2 subsamples. Next, we conducted item analysis and exploratory factor analysis (EFA) on sample 1 (*N* = 354) and confirmatory factor analysis (CFA) on sample 2 using Mplus 7.4. (*N* = 354). The maximum likelihood estimation was used for missing data in CFA (Graham et al., 1996). On this basis, C-SACS was examined for cross-gender measurement invariance. Additionally, we used the Pearson correlation to test the convergent validity and the hierarchical regression to test the incremental validity. Lastly, we used R 4.0.5 to assess the internal reliability of the C-SACS (total sample *N* = 708), Cronbach alpha (α), and McDonald’s Omega (ω) for internal consistency were calculated using the R packages (“MBESS Package,” Dunn et al., 2014; Hayes et al., 2020). And we performed the test–retest reliability on the re-test sample (*N* = 217).

The following parameters were used to identify the model fit: *χ*^2^, CFI (Comparative Fit Index), TLI (Tucker-Lewis index), RMSEA (Root Mean Square Error of Approximation), and SRMR (Standardized Root Mean Square Residual). The values of CFI and TLI >0.90 were considered a good model fit and the values >0.80 were considered an acceptable fit (McDonald and Ho, 2002). The values of RMSEA and SRMR <0.08 were considered an acceptable fit and <0.06 were considered an excellent fit (Marsh et al., 2004). The reference values of the *α* and *ω* are categorized as the following <0.50, 0.50 to <0.60, 0.60 to <0.70, 0.70 to <0.80, 0.80 to 0.90 and >0.90 which indicates unacceptable, poor, questionable, acceptable, good, and excellent internal consistency (Gliem and Gliem, 2003).

## Results

### Item analysis

First, we ranked the total score of C-SACS of sample 1 (*N* = 354) from high to low and divided the sample into a high-score group (i.e., 27% of the top ranking) and low-score group (i.e., 27% of the bottom ranking). According to the result of the t-test, the difference was significant (*p* < 0.001), indicating that items in each group had good degrees of discrimination. Based on the results of the correlation analysis, the correlation coefficient between each item and the total score ranged from 0.66 to 0.79 (*p* < 0.001), suggesting that each item had a good consistency with the scale (See Table 1).

**Table 1:** Item analysis (*N* = 354).

Items	Descriptive statistics		*t*-test		Correlation analysis (*r*)	
	*M*	SD	*t*	*p*	I-T_S_	I-T
1	4.97	1.16	13.90	<0.001	0.76^***^	0.70^***^
2	5.11	1.05	14.84	<0.001	0.79^***^	0.73^***^
3	5.32	1.01	14.88	<0.001	0.76^***^	0.73^***^
4	5.18	1.04	15.55	<0.001	0.83^***^	0.79^***^
5	4.90	1.13	11.24	<0.001	0.74^***^	0.66^***^
6	4.84	1.04	11.65	<0.001	0.80^***^	0.71^***^
7	5.17	1.00	12.95	<0.001	0.74^***^	0.75^***^
8	5.02	1.05	12.52	<0.001	0.81^***^	0.73^***^
9	4.94	1.07	11.52	<0.001	0.72^***^	0.68^***^
10	5.06	0.98	11.44	<0.001	0.81^***^	0.71^***^

M, mean; SD, standard error; I-T_S_ is the correlation between an item with subscale score, I-T is the correlation between an item with C-MPFI total scores; ^***^p < 0.001.

### Exploratory factor analysis

We performed an EFA on sample 1 (*N* = 354). First, we conducted the KMO sample fitness test and Bartlett sphericity test, and the results showed that the data were suitable for performing EFA (KMO = 0.90, *χ*^2^ = 1650.38, *df* = 45, *ρ* < 0.001). Next, components were rotated using the oblique direct oblimin procedure. Based on the criterion that the eigenvalue should be greater than 1, the results indicated a 2-factor model with a 61.81% cumulative variance explanation rate and the factor loading being between 0.56 and 0.94 for each item. Furthermore, based on the criterion that the absolute value of the cross-factor loading difference should be greater than 0.2 (Tabachnick et al., 2007), We deleted item 7 (“There is a basic sense I have of myself that does not change even though my thoughts and feelings do”) because its loadings on each factor were lower than 0.5 and its absolute difference was less than 0.2. We performed the EFA again, which suggested suitable data for performing EFA (KMO = 0.89, *χ*^2^ = 1650.38, *df* = 36, *ρ* < 0.001). Again, components were rotated using the oblique direct oblimin procedure. Based on the criterion that the eigenvalue should be greater than 1, the results indicated a 2-factor model (the eigenvalues were 4.01 and 3.78 respectively) with a 62.97% cumulative variance explanation rate and the factor loading being between 0.57 and 0.94 for each item. The two factors were named Centering and Transcending. The factor loadings, numbers, and names were consistent with the original SACS scale except for the removal of item 7 (See Table 2).

**Table 2:** Factor loadings for exploratory analysis of SACS.

Items	Factor 1	Factor 2
1. When I am upset, I am able to find a place of calm within myself.	**0.69**	0.10
2. I have a perspective on life that allows me to deal with life’s disappointments without getting overwhelmed with them.	**0.79**	0.06
3. Despite the many changes in my life, there is a basic part of who I am that remains unchanged.	0.22	**0.59**
4. As I look back upon my life so far, I have a sense that part of me has been there for all of it.	0.14	**0.73**
5. I allow my emotions to come and go without struggling with them.	**0.71**	0.02
6. I am able to notice my changing thoughts without getting caught up in them.	**0.89**	0.08
8. Even though there have been many changes in my life, I’m aware of a part of me that has witnessed it all.	0.07	**0.88**
9. I am able to access a perspective from which I can notice my thoughts, feelings, and emotions.	0.10	**0.57**
10. When I think back to when I was younger, I recognize that a part of me that was there then is still here now.	0.14	**0.94**

Factor 1 = Centering. Factor 2 = Transcending; Item 7 has been deleted. The loads on the two factors are 0.36 and 0.48, respectively. Loadings value ≥ 0.50 are shown in bold.

### Confirmatory factor analysis

**Figure 1:**
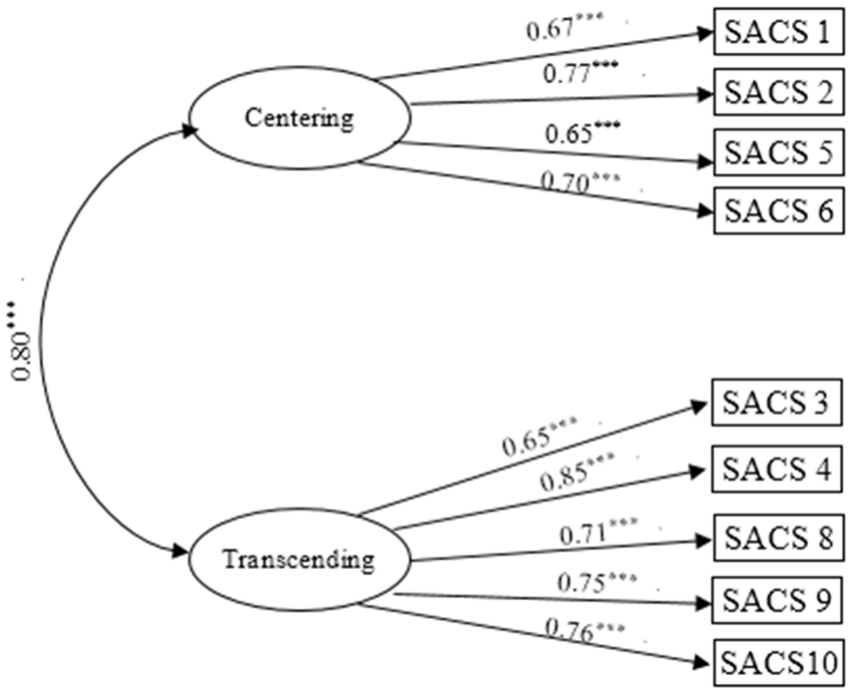
The 2-factor model (***Means *p* < 0.001).

We conducted CFA on sample 2 (*N* = 354) and constructed multiple models to validate the rationale of having a 2-factor model obtained in EFA. A single factor model with 10 items was constructed at first with poor model fit (*χ*^2^ = 165.71, *df* = 31, CFI = 0.866, TLI = 0.827, RMSEA = 0.135, SRMR = 0.059). In order to improve the model fit, a two-factor model with 9 items was constructed, which showed improved and good model fit (*χ*^2^ = 55.40, *df* = 22, CFI = 0.977, TLI = 0.963, RMSEA = 0.065, SRMR = 0.032). As shown in Figure 1, the loadings of the 9 items on the factor were between 0.65 and 0.80, and the correlation of “Centering” and “Transcending” was *r* = 0.80.

### C-SACS measurement invariance model

The complete sample (*N* = 708) was analyzed for measurement equivalence, and a multi-group model was used to explore whether the C-SACS has equivalence across gender (Table 3). In the Configural invariance model, the model fit was acceptable and met the conditions of the next equivalence analysis. We also set the factor loading equivalence (metric invariance model), the index intercept equivalence (scalar invariance model), and the error variances equivalence (strict invariance model) based on the previous model. The results showed that the ΔCFI and ΔRMSEA between the Configural and the metric invariance model, between the metric and the scalar invariance model, and between the scalar invariance model and the strict invariance model were all less than 0.01. The results indicated that the Configural invariance model, the metric invariance model, the scalar invariance model, and the strict invariance model were reliable cross-gender (Cheung and Rensvold, 2002; Chen, 2007).

**Table 3:** Model fit statistics and invariance testing across gender (*N* = 708).

Model	SB*χ*^2^	*df*	CFI	TLI	SRMR	RMSEA [90%CI]	ΔCFI	ΔRMSEA
Male	37.97	22	0.972	0.954	0.038	0.068(0.028,0.103)		
Female	93.69	22	0.968	0.948	0.034	0.077(0.061,0.093)		
Configural model	131.65	44	0.969	0.949	0.035	0.075(0.060,0.090)		
Metric invariance	138.54	51	0.969	0.956	0.046	0.070(0.056,0.084)	0.001	0.005
Scalar invariance	157.03	58	0.965	0.957	0.054	0.069(0.057,0.083)	0.004	0.001
Strict invariance	173.39	59	0.960	0.951	0.062	0.064(0.060,0.069)	0.005	0.005

### Convergent validity

We used the Pearson correlation analysis to evaluate its convergent validity. The results suggested that the Transcending score was not significantly correlated with the CFQ-F score. The SACS total score and its respective factor scores were found to be significantly negatively correlated with DASS-anxiety, stress, depression, AAQ-II, and CFQ-F scores, and significantly positively correlated with SWLS, POM, and MAAS scores (Table 4).

**Table 4:** Correlations for the SACS with other scales (*N* = 343).

	Variables	Centering	Transcending	SACS total score
Positive indicators	SWLS	0.44^***^	0.36^***^	0.42^***^
	POM	0.41^***^	0.24^***^	0.34^***^
Negative indicators	DASS	−0.34^***^	−0.19^***^	−0.26^***^
	DASS-Anxiety	−0.30^***^	−0.18^***^	−0.25^***^
	DASS-Stress	−0.32^***^	−0.15^**^	−0.24^***^
	DASS-Depression	−0.34^***^	−0.20^***^	−0.27^***^
ACT (other domains)	AAQ–II	−0.29^***^	−0.10^*^	−0.20^***^
	CFQ–F	−0.29^***^	−0.07	−0.18^***^
	MAAS	0.37^***^	0.24^***^	0.32^***^

^*^Means *p* < 0.05; ^**^Means *p* < 0.01; ^***^Means *p* < 0.001.

### Incremental validity

To examine the different predictive power of self-as-context and other ACT processes (experiential avoidance, cognitive fusion) on negative emotional indicators (anxiety, stress, depression) and mental health indicators (life satisfaction, affective well-being), we used the hierarchical regression method to test its incremental validity. We treated the SWLS, POM, DASS-Anxiety, DASS-Stress, and DASS-Depression scores as the dependent variables, the AAQ-II and CFQ-F scores as the first layer of the regression model, and the SACS Centering and Transcending scores as the second layer of the model. As suggested in Table 5, by controlling for the effects of AAQ-II and CFQ-F on dependent variables, the impacts of the SACS Centering and Transcending subscales (ΔR^2^) on SWLS, POM, DASS-Anxiety, DASS-Stress, and DASS-Depression remained significant. The results indicated that self-as-context, experiential avoidance, and cognitive fusion are all core processes of ACT. Additionally, self-as-context is different from the other two. It uniquely predicted negative emotions, life satisfaction, and affective well-being.

**Table 5:** Incremental validity of SACS.

Dependent variable		Independent variable	*β*	*R* ^2^	Δ*R*^2^
SWLS	First layer	AAQ–II	−0.23^**^	0.17^***^	
		CFQ–F	−0.22^**^		
	Second layer	Centering	0.20^**^	0.29^***^	0.12^***^
		Transcending	0.18^**^		
POM	First layer	AAQ–II	−0.34^***^	0.30^***^	
		CFQ–F	−0.24^***^		
	Second layer	Centering	0.25^***^	0.36^***^	0.06^***^
		Transcending	0.02		
DASS–Anxiety	First layer	AAQ–II	0.49^***^	0.37^***^	
		CFQ–F	0.15^*^		
	Second layer	Centering	−0.1	0.38^***^	0.01^*^
		Transcending	−0.02		
DASS–Stress	First layer	AAQ–II	0.39^***^	0.39^***^	
		CFQ–F	0.28^***^		
	Second layer	Centering	−0.16^**^	0.41^***^	0.02^**^
		Transcending	0.04		
DASS–Depression	First layer	AAQ–II	0.50^***^	0.35^***^	
		CFQ–F	0.13^*^		
	Second layer	Centering	−0.17^**^	0.38^***^	0.03^**^
		Transcending	−0.01		

^*^Means *p* < 0.05; ^**^Means *p* < 0.01; ^***^Means *p* < 0.001.

### Reliability analysis

The C-SACS (9 items) showed acceptable to good internal consistencies in the total scale and subscales (i.e., Centering and Transcending): total scale *α* = 0.88[0.87, 0.90], McDonald’s omega (*ω*) = 0.88[0.87, 0.90]; Centering subscale *α* = 0.80[0.78, 0.83], *ω* = 0.80 [0.78, 0.83]; Transcending *α* = 0.85 [0.83, 0.88], *ω* = 0.85 [0.83, 0.88]. Additionally, we tested the retest reliability of C-SACS and found the test–retest reliability (ICC) was. 0.73 [0.64, 0.79] and 0.72[0.64, 0.79] respectively.

## Discussion

The results of the item analysis showed that items in the Chinese version of SACS had good degrees of discrimination, with each item being highly consistent with the total score. The results of the exploratory factor analysis suggested a two-factor model (Centering and Transcending). Except for item 7, the factor loading of each item was above 0.5. After removing item 7 (“There is a basic sense I have of myself that does not change even though my thoughts and feelings do”), the cumulative variance explained rate increased to 62.97%. While retaining sufficient content and internal consistency, we can improve accuracy and usability, and obtain a more stable factor structure of the scale by excluding items that can be omitted and reducing the corresponding response burden (Francis et al., 2016).

The confirmatory factor analysis results also suggested that the C-SACS two-factor model (9 items) fit better than the one-factor model (10 items), and was consistent with the original scale in terms of structure and content (Zettle et al., 2018). Centering reflects the ability to calmly deal with various negative thoughts and experiences while Transcending reflects the invariable characteristics of the observing self, not being disturbed by various internal negative thoughts and feelings, and can observe reality objectively. Therefore, the C-SACS two-factor structure has good stability. Based on the confirmatory factor analysis, we constructed multiple models (i.e., the configural model, the metric invariance model, and the scalar invariance model) to examine the measurement equivalence of the C-SACS across genders on the entire sample (*N* = 708). The configural model was established, indicating that the C-SACS has the same composition of latent variables in males and females, and the next equivalence test could be carried out. The metric invariance model was established, showing that the factor loading of the C-SACS in males and females was equivalent and that the observed variable would change consistently with the latent variables in different groups. The scalar invariance model was established, indicating that the intercepts of the scale were equivalent in each group, and either group had the same reference point. The strict invariance model was established, indicating that error variance across genders was equivalent. Therefore, the C-SACS scores were comparable across genders. Moreover, the results from the reliability analysis also showed that the C-SACS and all subscales had high internal consistency and test–retest reliability. These findings suggested the reliability and stability of the C-SACS.

Based on the correlation analysis of the C-SACS total score and its two subscales, we found that the C-SACS total score and its subscale scores were significantly positively correlated with positive indicators of individual mental health (life satisfaction and affective well-being). This result was consistent with previous studies (Francis et al., 2016; Moran et al., 2018), indicating that the higher the level of self-as-context, the more flexible the person is capable of coping with negative experiences in life, leading to higher levels of subjective well-being. The study also found that the C-SACS total score and its subscale scores were significantly negatively correlated with the negative indicators of mental health (negative emotions such as anxiety, stress, and depression), which was also consistent with previous studies (Godbee and Kangas, 2020; Østergaard et al., 2020), suggesting that the lower the level of self-as-context, the more likely the person is going to be troubled by various negative emotions and experiences in daily life. The current study also examined the relationship among the C-SACS total score, its subscale scores, and other core processes of psychological flexibility (experiential avoidance, cognitive defusion, and present moment awareness). ACT theories believe that psychological flexibility is composed of multiple interrelated but distinct core processes (Hayes et al., 2006). The results indicated that the C-SACS total score and its subscale scores were significantly negatively correlated with experiential avoidance and cognitive fusion. The C-SACS total score and its subscale scores were also significantly positively correlated with the present moment awareness (mindful attentional awareness), which is consistent with the ACT theory and the original scale (Hayes et al., 2006; Zettle et al., 2018), indicating that the Chinese version of SACS has good convergent validity.

In addition, the study also found that self-as-context, one of the core processes of psychological flexibility, has good predictive power for both positive and negative indicators of mental health, which is consistent with previous research findings (Moran et al., 2018; Zettle et al., 2018; Godbee and Kangas, 2020). Through hierarchical regression analysis, self-as-context was good at predicting life satisfaction, affective well-being, and negative emotion, which is different from other processes of ACT (i.e., experiential avoidance and cognitive defusion). This also indicates that SACS has good validity and unique predictive power. Therefore, this effective self-as-context assessment tool can provide ACT researchers and clinicians with another specific sub-process measure, promoting the related research on self-as-context and ACT in the field of mental health, and could potentially offer new insights and references for mental health interventions.

There were some limitations of this study that should be noted. Our sample only consisted of university students, which may limit the external validity of the current study. We are hopeful that future research will be able to find generalized results and impacts across different Chinese populations. Another limitation was that fewer males participated in our study, which may lead to sample bias. Future research should focus more on balancing the ratio bias between males and females. Furthermore, our sample data were collected from non-clinical populations. With the use of the original SACS, the previous study has shown a positive impact of self-as-context on mediating functional improvement in a clinical sample (Yu et al., 2017). Additional research needs to be conducted to explore the overall applicability of C-SACS in the clinical environment as well.

In conclusion, the factor structure of SACS tested in Chinese college students was consistent with the original scale. All of the items had reached the standard psychometrics with good reliability, validity, and unique predictive power, and can be used to evaluate the levels of self-as-context among Chinese college students.

## Data availability statement

The datasets generated during and/or analyzed during the current study are available from the corresponding author upon reasonable request.

## Ethics statement

This study was approved by the Ethical Committee of Anhui Normal University. The participants provided their written informed consent to participate in this study.

## Author contributions

SF conceived and designed the analysis, collected the data, performed the analysis, and wrote the manuscript. MH supported the data collecting and assisted in data analysis. YW supported writing. All authors contributed to the article and approved the submitted version.

## Funding

This study was supported by Philosophy and Social Science Planning key Project of Anhui Province (grant number AHSKZ2020D37).

## Acknowledgments

We would like to thank the support of Anhui Provincial Women’s Federation and Anhui Provincial Department of Education 2022 Annual Women’s Theory Research Key Project (2022-FNYJ-002), Open Fund Project of Key Laboratory of Philosophy and Social Science of Anhui Province on Adolescent Mental Health and Crisis Intelligence Intervention, and Anhui Topnotch Talents of Disciplines in Universities and colleges (SF).

## Conflict of interest

The authors declare that the research was conducted in the absence of any commercial or financial relationships that could be construed as a potential conflict of interest.

## Publisher’s note

All claims expressed in this article are solely those of the authors and do not necessarily represent those of their affiliated organizations, or those of the publisher, the editors and the reviewers. Any product that may be evaluated in this article, or claim that may be made by its manufacturer, is not guaranteed or endorsed by the publisher.

## Appendix 1

The Chinese version of the SACS used in the study.
